# Prophylactic effect of topical (slow-release) and systemic curcumin nano-niosome antioxidant on oral cancer in rat

**DOI:** 10.1186/s12906-022-03590-5

**Published:** 2022-04-19

**Authors:** Behzad Fazli, Soussan Irani, Hassan Bardania, Mahdieh-Sadat Moosavi, Bita Rohani

**Affiliations:** 1 Student Research Committee, Faculty of Dentistry, Aja University of Medical Sciences, Tehran, Iran; 2 Dental Research Centre, Oral Pathology Department, Dental Faculty, Hamadan University of Medical Sciences, Hamadan, Iran; 3 Lecturer at Pathology Department of Faculty of Medicine, Griffith University, Gold Coast, Australia; 4 Cellular and Molecular Research Center, Yasuj University of Medical Sciences, Yasuj, Iran; 5 Dental Research Center, Dentistry Research Institute, Department of Oral and Maxillofacial Medicine, Faculty of Dentistry, Tehran University of Medical Sciences, Tehran, Iran; 6 Department of Oral Medicine, Faculty of Dentistry, Aja University of Medical Sciences, Tehran, Iran

**Keywords:** Antioxidant, Curcumin, Niosome, Oral cancer, Oxidant

## Abstract

**Background:**

Oral malignancies have a significant effect on the quality of life among the affected patients. Curcumin is an antioxidant with a low bioavailability in the target tissue. Niosomes are carriers of increasing the therapeutic effects of drugs and reducing their side effects. This study aimed to determine the effective dose of curcumin niosome in the culture and then to compare its prophylactic effect in the form of mouthwash with that of its injectable form on oral cancer in rats.

**Methods:**

This was an in-vitro and in-vivo study. Firstly, KB oral cancer cells and human umbilical vein endothelial cells (HUVEC) were treated in separate groups with free curcumin, curcumin-loaded niosomes, and the unloaded niosomes at four doses of 4, 8, 16, and 32 μg. The study rats were then divided into the following four groups: 1) no intervention, 2) only carcinogenic substance, 3) carcinogenic substance with curcumin-loaded niosome injection, and 4) carcinogenic substance with a mouthwash containing niosome.

**Results:**

At the cellular level, a dose of 16 μg after 24 h was selected as an effective dose. In the animal phase, the use of injectable curcumin niosome was observed to significantly prevent the development of severe forms of dysplasia.

**Conclusions:**

In this in-vitro and in-vivo study, curcumin-loaded niosome was effective in preventing the development of severe forms of dysplasia and the inhibition of the growth of cancer cells.

## Background

Oral cancer is a broad term that encompasses all different diagnoses of malignancies occurring in oral tissues. Squamous cell carcinoma (SCC) accounts for more than 90% of oral malignancies worldwide. Accordingly, it may occur in lips, labial and buccal mucosa, tongue, retromolar pad, the floor of the mouth, gums, and hard palate [1]. As well, malignancies in the oropharynx, hypopharynx, nasopharynx, tonsils, larynx and salivary glands are known as oral cancer. Of note, oral SCC is multifactorial, and many internal and external factors play a role in its occurrence, including smoking, non-smoking tobacco, alcohol, having contact with occupational factors (environmental pollutants), radiation, vitamin and mineral deficiencies, nutritional factors, carcinogenic bacteria, candida fungus, oncogenic viruses, and immunosuppression. According to recent estimations, oral cancer is the thirteenth most common cancer worldwide. Although the incidence of oral cancer varies in different parts of the world, its prevalence in almost all regions around the globe in women is twice that of in men. According to the latest World Health Organization estimations, in 2020, about 15 million people were diagnosed with new cases of cancer [2].

The treatment of oral SCC is determined by the grade of the disease, including extensive surgery, radiation therapy, chemotherapy, or a combination of them. Conventional treatments cause more side effects, and in the best condition, they can only increase the life span in patients for several years. Despite several advances in the treatment and understanding of the molecular pathogenesis of oral cancer, survival rates have not improved significantly in recent decades and remained between 50 and 59 percent. In recent years, the use of natural compounds due to their low side effects, low costs, and promising effects has been considered to fight cancer. Nowadays, many herbal compounds with different biological effects, which are effective in the treatment of various cancers, have been isolated and introduced to modern pharmaceutical science [1].

Recently, several researchers in their studies have shown the roles of free radicals and reactive oxygen species (ROS) in several pathological cases, including different types of cancer. These oxidative mediators of oxygen attack DNA and other cellular components such as lipids and proteins, and consequently cause a series of reactions that in turn can damage DNA double-strands [3]. The accumulation of DNA damage due to lack of repair or incomplete repair can subsequently lead to mutagenesis and cancerous changes. The harmful effects of ROS are also balanced by non-enzymatic and enzymatic antioxidants [4]. Additionally, it was indicated that loss of antioxidant defense mechanisms leads to oxidative damage to cells and normal tissues. The impaired delicate balance between oxidants/antioxidants in the body plays an important role in carcinogenesis. The increased levels of oxygen free radicals (ROS), reactive nitrosative free radicals (RNS), or both of them as well as the decreased antioxidants are seen in various types of cancer, including head and neck cancers [5, 6]. The relationship between free radicals and disease was firstly described in detail based on the concept of "oxidative stress" by Sies. Accordingly, he defined oxidative stress as an imbalance between oxidants and antioxidants in favor of oxidants, which could potentially lead to damage [7].

An antioxidant is any molecule with the ability to stabilize or inactivate free radicals [5]. Low levels of antioxidants were indicated to be associated with an increased risk of developing oral cancer. Various studies have previously shown a lack of antioxidants or a decrease in antioxidant capacity in blood samples and tissues of patients with oral cancer and precancerous lesions [8]. Antioxidants also help the immune system in responding more strongly. Notably, antioxidants have the potentials to prevent, inhibit, and reverse various stages involved in oral cancer [9].

Curcumin, as a hydrophobic polyphenol and an active ingredient in turmeric, is derived from the rhizome of the turmeric plant with the Latin name Turmeric is scientifically named *Curcuma longa*. Correspondingly, it has a wide range of biological and pharmacological activities, and is, in fact, a powerful antioxidant. The only limiting factor regarding the use of this substance is its low solubility and consequently its low stability and bioavailability in the human body. Therefore, to provide the expected therapeutic effects of this substance, it must be used frequently in large quantities [10].

Nanotechnology can be used to create nanometer-sized drugs. Drug delivery systems are also developed to improve the pharmacological and therapeutic properties of drugs administered for these patients. In this study, we used niosome nanocapsules, which are carriers formed from the accumulation of non-ionic surfactants in an aqueous medium, and then create a vesicle-like bilayer structure. Moreover, they have two parts, including a hydrophilic segment and a hydrophobic segment. The use of drug-containing niosomes improves the pharmacokinetics of the administered drugs, which consequently increases the therapeutic effects of the drug and reduces its side effects. Due to the drug’s retention within the niosome, drug stability can also be increased [11].

Chemoprevention of cancer referred to agents with low side effects and toxicity, which can neutralize a carcinogen substrate. Natural components are much more biocompatible than synthetic products. These compounds can be effective in apoptosis, cellular immortalizing, proliferation, carcinogenesis of infecting factors, genomic stability, change in cellular metabolism, immune escaping, invasion, metastasis, and angiogenesis [12]. The preferable route of administration is being investigated yet.

Based on the above-mentioned statements, it seems that the disadvantages of curcumin can be resolved using the niosome system. The main idea of this study firstly was to obtain the effective dose of curcumin niosome in the culture medium of KB oral cancer cell lines (human epithelial carcinoma cells as a model of cancer cells), and then to investigate the prophylactic effect of this drug in the mouthwash form (topical) on the induced oral cancer in the rat. Finally, it was aimed to compare its effect with that of the injectable (systemic) form of the drug.

## Methods

This basic-applied research was approved by the ethics committee of Aja University of Medical Sciences (Ethics ID: IR.AJAUMS.REC.1398.180). All the experiments were performed in terms of the relevant guidelines and the study was reported in terms of the ARRIVE guidelines (Animal Research: Reporting of In Vivo Experiments). As well, the study was done on the following two levels:

### At the cellular level (in vitro)

#### Niosome preparation

Niosomes containing curcumin were prepared by the thin-film hydration method [13]. Briefly, Tween 80, Span 80; cholesterol with molar ratio 35, 35, 30 were dissolved in chloroform. Then, curcumin (0.5 mg/mL-1 in methanol) was added to the mixture in a round-bottom flask and dried under the vacuum in a rotary evaporator at 40 °C and 150 rpm. The resulting thin film was hydrated by 2 ml of phosphate buffer saline and sonicated using UP400S Ultrasonic processor (Hielscher, Germany) four times (Cycle 0.5, Amplitude 60%), each for 45 s with an internal rest of 30 s to make unilamellar vesicles.

Niosomes were characterized by dynamic light scattering DLS analysis (Nanoparticle Analyzer (Horiba SZ-100). The scattering was recorded at a backscattering angle of 173 and 25 °C.

In this basic-applied research, the HPLC device (A Eurospher 100–5 C18 column (200 4.6 mm, 5 lm, KNAUER, Germany)) was firstly used to determine the amount of the loaded curcumin. The mobile phase consisted of a mixture of acetonitrile and water (containing 5% acetic acid) 55:45 (v/v), with a constant 15.1 mL/ min flow rate; detection was performed at 425 nm. Thereafter, the amount of the loaded drug was calculated compared to its initial amount and all the experiments on curcumin were performed in terms of the relevant guidelines and regulations.

Cells’ groups were considered as follows (the cells were obtained from the cell bank of Pasteur Institute, Tehran, Iran, and curcumin (CB0346) were purchased from Bio Basic Inc. (Canada)):

1. The control group (HUVEC cells) treated with free curcumin
2. The control group (HUVEC cells) treated with niosome with no curcumin
3. The control group (HUVEC cells) treated with both curcumin and niosome
4. The KB cell group treated with free curcumin
5. The KB cell group treated with niosome with no curcumin
6. The KB cell group treated with both curcumin and niosome

The KB and HUVEC cells were cultured in the Dulbecco’s modified Eagle’s Medium (DMEM) medium supplemented with 10% FBS and 1% antibiotics (streptomycin/penicillin) (flask 25 cm^2^). After 24 h, the supernatant was completely removed. After trypsinization, the cells (8 × 10^3^ cells/well) were transferred to a 96-well plate and then incubated for 24 h in a humidified incubator under 5% CO_2_, at a temperature of 37 °C. At the first stage, 100 µl of four final concentrations of the drug (32, 16, 8, and 4 μg/ml curcumin in DMEM medium) were treated in each one of the four horizontal wells in each group. Of note, these four concentrations were repeated three times. The plates containing the cells and drugs were incubated for 24 h under the same conditions. By passing the desired time, 96-well plates were removed from the incubator and the culture medium was then removed. Subsequently, the cells were washed using phosphate buffer, followed by the addition of (3- (4,5-dimethyltetrazolyl -2-)—2,5-diphenyltetrazolium bromide) MTT solution (with 0.5 mg/ml of MTT in phosphate buffer) to each well and then incubated for 3 h at 37 °C. Afterward, 100 μl of DMSO (Dimethyl sulfoxide) was substituted for MTT medium and then kept for 30 min in the darkness. DMSO was used to dissolve the produced formazan crystals and after that, these crystals formed a purple dye, indicating the bioavailability of the treated cells. The optical absorption rates of the plates were measured at 490 nm and 570 nm using an ELX800 UV universal microplate reader (Bio-Tek Instruments Inc., Vermont, USA). The above-mentioned method was repeated for each group after 48 and 72 h. In addition, the bioavailability or percentage of survival of the cells affected by the concentration of niosomes was calculated by dividing the optical absorption of the treated wells by the optical absorption of the control group multiplied by 100. The concentration of niosome, which can reduce the percentage of cell life by half, was considered as IC50 (half of the maximal inhibitory concentration). Thereafter, the effect of six intervention groups on cells was investigated using the MTT assay. As well, three replicates were given for each group. Additionally, averages were taken from the repetitions and their mean was compared statistically. In other words, the MTT results were expressed as a percentage of viability. Finally, one-way ANOVA and Duncan post hoc tests were used to analyze experimental results to determine whether the differences are statistically significant.

### At the level of laboratory work on rats

**Fig. 1:**
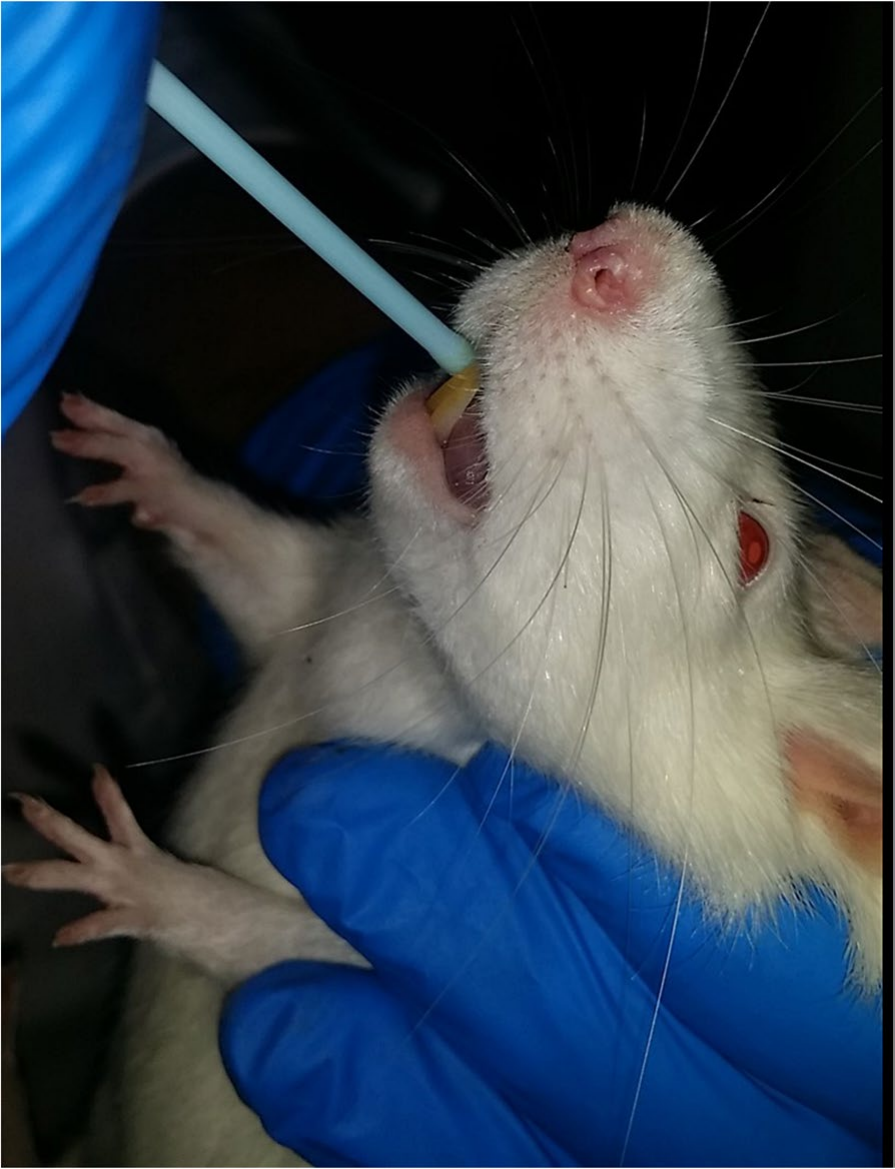
Mouthwash was applied to the entire oral cavity using an applicator

Laboratory work on rats was performed at the Pharmacy Faculty of Tehran University of Medical Sciences for a duration of 10-month. All the ethical considerations and the protocols related to work on animals were under the supervision of the Animal Care and Use Committee (ACUC) of this center and all the methods were also performed in terms of the relevant guidelines and regulations. In this study, 32 Sprague Dawley male rats in the age range of 3 to 3.5 months with approximately the same weight, were used. Accordingly, they were randomly divided into 4 groups (Confidence Interval 95%). Thereafter, the animals were placed in special cages with a bed of sawdust. The type and feeding conditions were similar for all the study groups and they were in a cycle of 12 h of lightness/darkness. Moreover, the room temperature (23 ± 2 ˚C) was the same and they had free access to water. To induce oral carcinoma, 4-Nitroquinoline-1-Oxide (4NQO) provided by the Sigma German company, was used. For every kilogram of rat weight, 30 ppm of 4NQO solution was prepared and daily used for each cage. To prepare this amount of solution, a digital sensitive scale was used and for animals’ consumption, the solution was placed in foil-covered bottles to prevent the light’s effects [14]. The four study groups were as follows:

- Group 1: the negative control group (this group received neither carcinogenic substances nor drugs).
- Group 2: the positive control group (the carcinogenic substance was only used in this group for 38 weeks).
- Group 3: In this group, besides receiving carcinogenic substances for 38 weeks, 4 mg/kg of curcumin was injected intravenously every 48 h. The injection dose was 0.5 ml.
- Group 4: In this group, for 38 weeks, besides receiving carcinogenic substances, curcumin niosome mouthwash was used three times daily (with an interval of 8 h), each time for 5 min. Mouthwash was applied to the entire oral cavity using an applicator (ear cleaner). After applying mouthwash, the rats were prevented from having free access to water and food for 5 min (Fig. 1). Finally, the rats were sacrificed by dislocation in the cervical vertebrae. The obtained tongue tissue samples were fixed in Neutral buffered Formalin for 24–48 h before machine processing and then cut by 4 mm thickness. Next, the sections were stained using the Periodic acid Schiff (PAS) method to assess the basement membrane integrity. Several sections were prepared from the anterior, middle and posterior portions of the tongue. The specimens were examined under the microscope (Olympus BX41 DIC Microscope, Tokyo, Japan), and the microscopic images were captured. Finally, all the sections were evaluated for dysplasia [15] and grading according to the Banoczy criteria [16]. Epithelial dysplasia was graded as mild when two of the mentioned histologic changes were present, moderate when three or four changes were noted, and severe when five or more of the changes were present. The sections were independently scored by two different persons. In instances where they disagreed, a face-to-face consensus review was done.

## Results

### At the cellular level (in vitro)

**Fig. 2:**
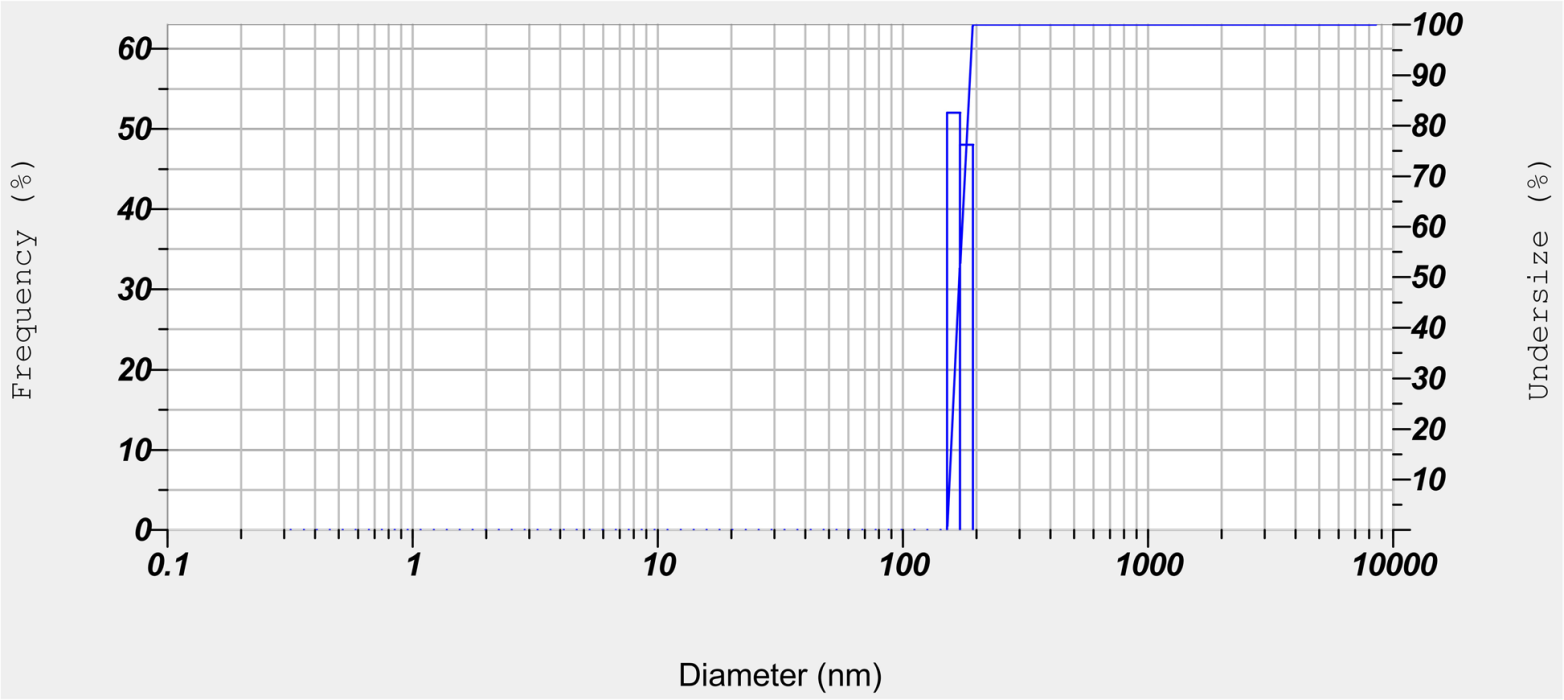
DLS analysis results for curcumin-loaded niosomes

The entrapment efficiency of curcumin in niosomes was calculated by comparing it with the un-encapsulated drug. The results showed that 98% of initial curcumin was loaded into niosomes. Niosomes were characterized by DLS analysis and its results showed that these nanostructures have about 171.2 ± 10.4 nm in size (Fig. 2).

**Fig. 3:**
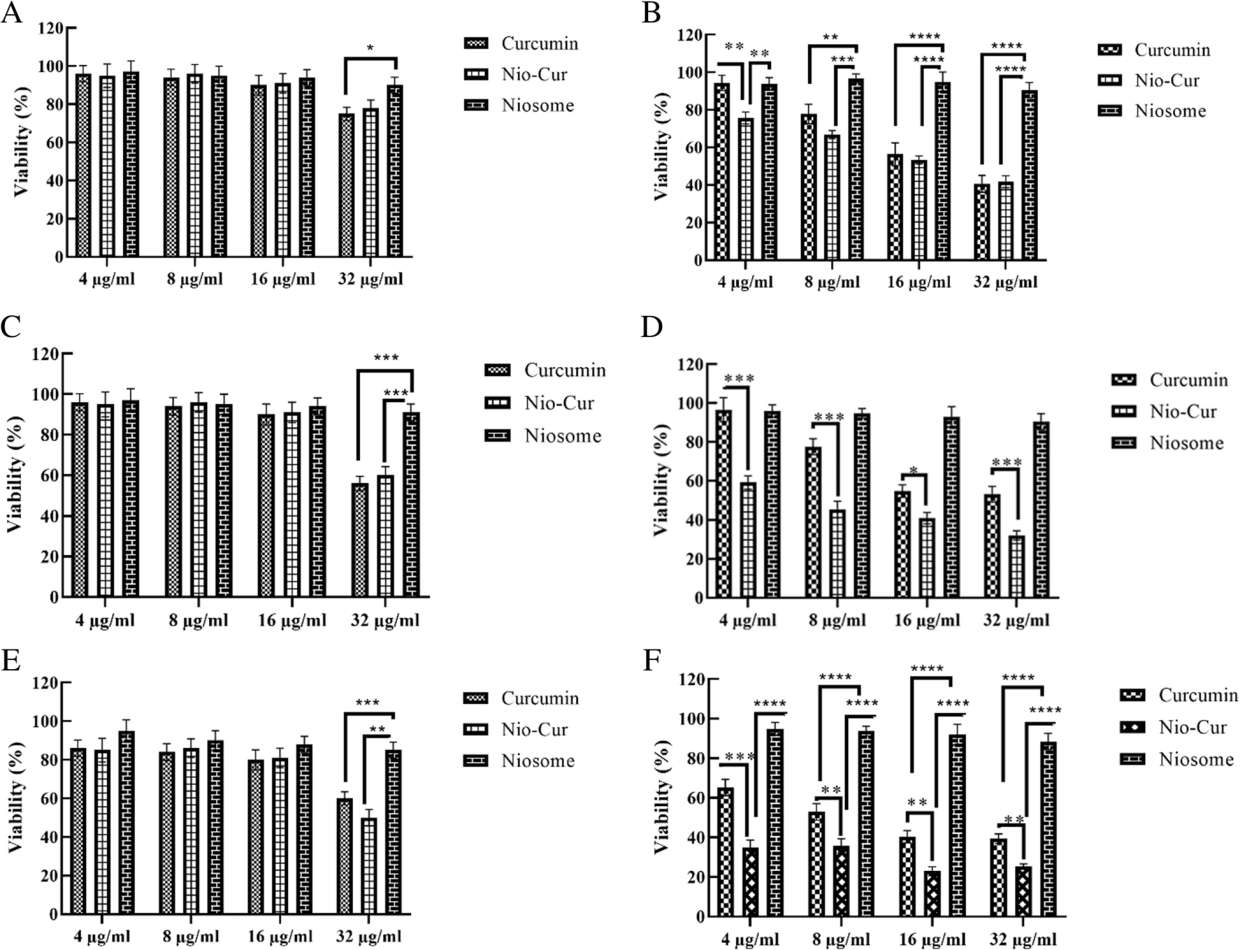
Evaluation of the toxicity effects of curcumin-loaded niosome, free curcumin, and unloaded niosome using MTT assay: **A** after 24 h in the control culture medium, **B** after 24 h in KB culture medium, **C** after 48 h in the control culture medium, **D** after 48 h in KB culture medium, **E** after 72 h in the control culture medium, and **F** after 72 h in the KB medium

The effect of cytotoxicity on KB oral cancer cells and HUVEC cells (control group) was investigated after 24, 48, and 72 h using the MTT method, and the results are shown in the following figures (Fig. 3).

According to diagram 2A, no significant changes were observed after 24 h in the control group (HUVEC cells) at any dose in the form of free curcumin, curcumin-loaded niosome, and unloaded niosome (*P* > 0.05).

As shown in diagram 2B, curcumin-loaded niosome had a more significant toxicity effect at a dose of 4 μg in the KB cell group compared to free curcumin after 24 h (*P* < 0.01), which is marked by two stars in the diagram. No significant changes were seen at other dosages.

As shown in diagram 2C, no significant changes were seen at any dose after 48 h in the control group (HUVEC cells) (*P* > 0.05).

Based on diagram 2D, the toxicity effect of curcumin-loaded niosome was highly significant in the KB cell group compared to that of free curcumin after 48 h at doses of 4, 8, and 32 μg (*P* < 0.0001), which is marked by three stars in this diagram. This was also significant at a dose of 16 μg (*P* < 0.01), which is marked by one star in this diagram.

According to diagram 2E, no significant changes were seen at any dose after 72 h in the control group (HUVEC cells) (*P* > 0.05).

As shown in diagram 2F, the toxicity effect of curcumin-loaded niosome was highly significant in the group of KB cells compared to free curcumin after 72 h at a dose of 4 μg (*P* < 0.0001). This significance was also seen in other doses with *P* < 0.001.

Because this study aimed to investigate the effect of using curcumin-loaded niosome in the form of mouthwash and injection, and according to the results of the above-mentioned analyses, the dose of 16 μg was selected as an effective dosage because it showed the best performance in 24 h and nearly 50% cell viability was maintained at this concentration.

### At the level of laboratory work on rats

To have a criterion for histopathological evaluations, a histopathological view was firstly prepared from the normal rat group in which no intervention was performed.

**Fig. 4:**
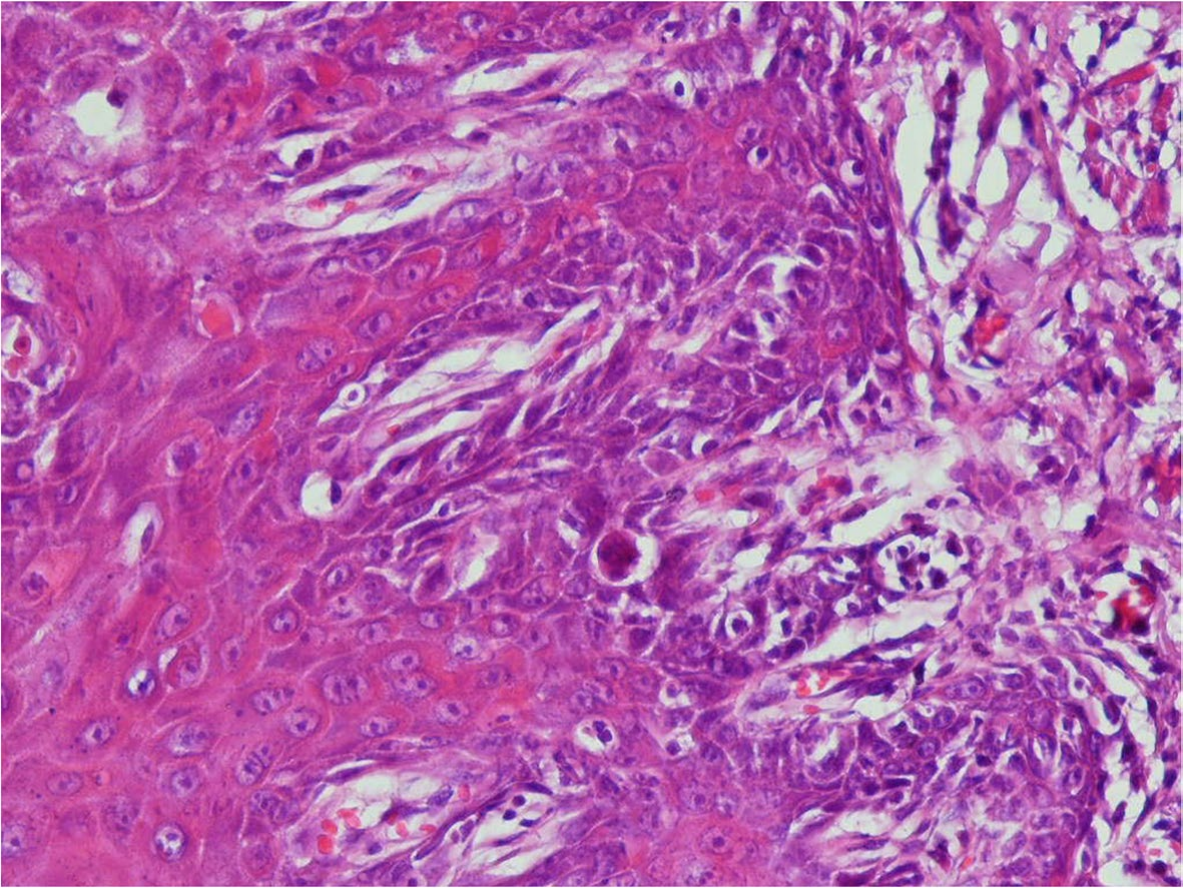
High power section of dorsal tongue mucosa of the carcinogen-treated rat shows individual cell keratinization, abnormal superficial mitosis, abnormal variation in cell and nuclear size and shape, and loss of polarity of basal cells. Underlying connective tissue shows angiogenesis (× 400)

In the following, histopathological images of the negative control group are placed for better comparison (Fig. 4).

**Fig. 5:**
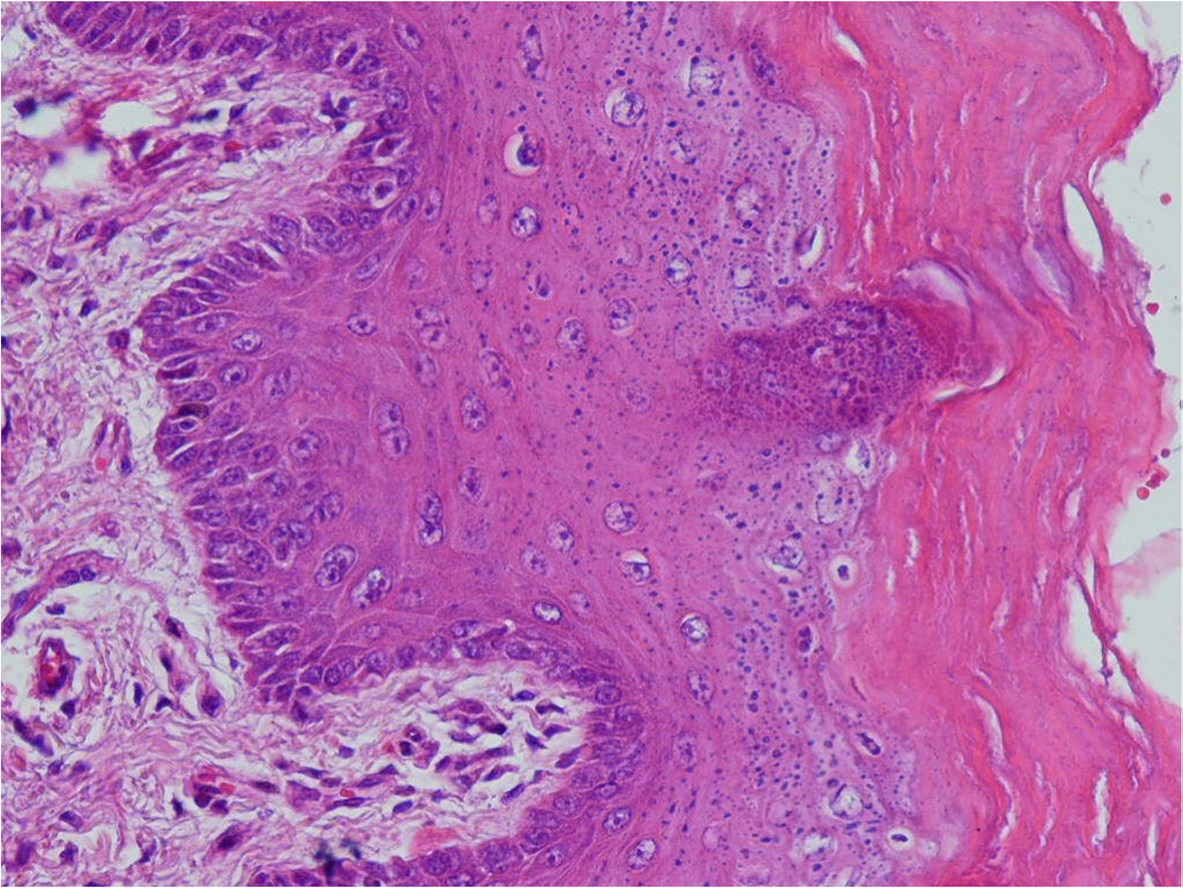
High power section of hyperkeratotic dorsal tongue mucosa of a rat treated with both carcinogen and curcumin mouthwash illustrates a relatively normal appearance compared to the samples that only received carcinogen (× 400)

**Fig. 6:**
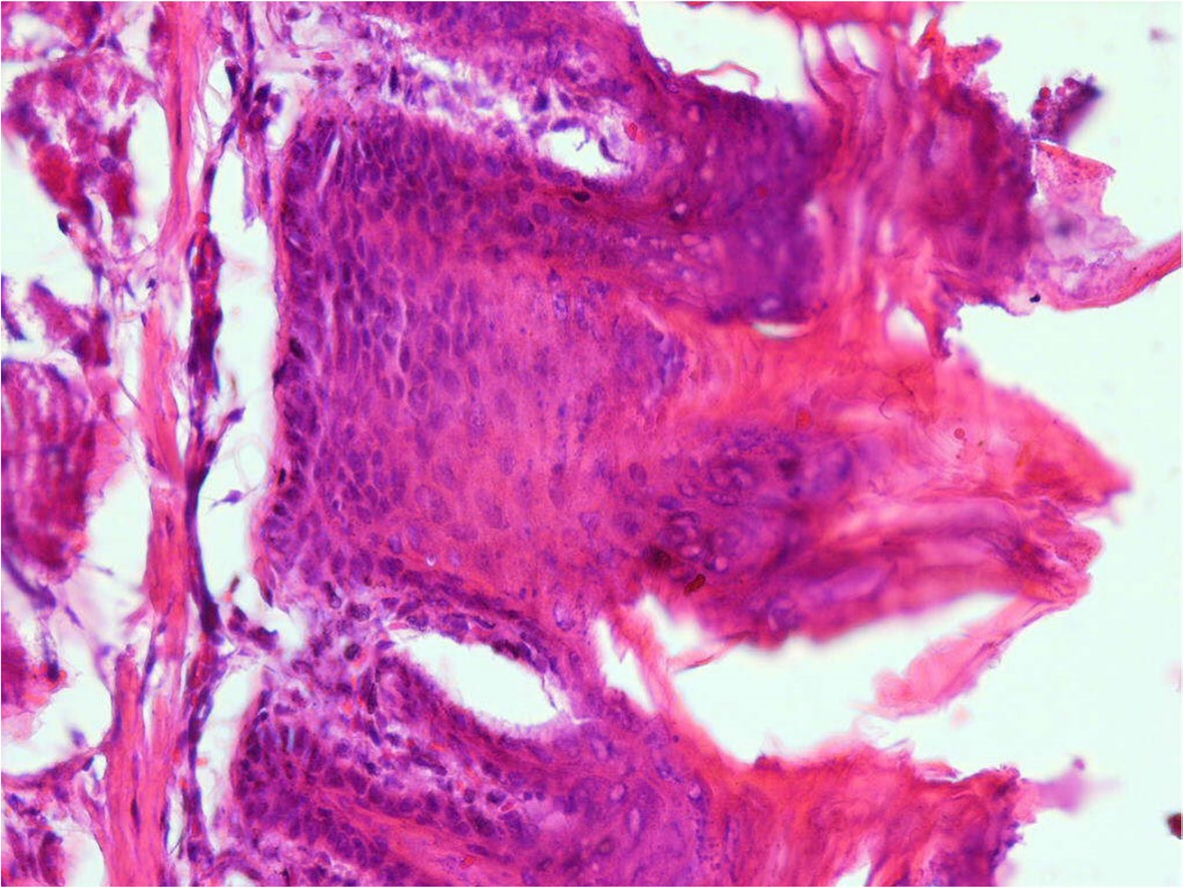
Low and high power sections of hyperkeratotic dorsal tongue mucosa of a rat treated with both carcinogen and systemic curcumin-loaded niosome show a relatively normal epithelium compared to the samples that only received carcinogen (× 400)

The following are the histopathological images of the mouthwash (Fig. 5) and injection (Fig. 6) groups.

Statistical analysis was performed after the histopathological evaluations of mucosal epithelial tissue for precancerous changes. According to the result of this analysis, the difference of several groups with the negative control group was evaluated using Fisher’s exact test (Table 1). The result indicated that the mouthwash group had no statistically significant difference with the negative control group; however, the intravenous injection group was able to prevent the development of severe forms of dysplasia significantly (*P* = 0.026). According to the microscopic view presented in the previous section as well as the evaluations performed, both mouthwash and injectable forms of curcumin-loaded niosome clinically and significantly improved the condition of the rats in confronting oral cancer’s development compared to the negative control group.

**Table 1:** Results of Fisher’s test statistical analysis

severe		moderate		mild		normal		groups
percent (*P-*value)	Number of criteria	percent (*P*-value)	Number of criteria	percent (*P*-value)	Number of criteria	percent (*P*-value)	Number of criteria	
0	0	0	0	0	0	100	8	Positive control
62.5	5	37.5	3	0	0	0	0	Negative control
37.5 (0.619)	3	50 (>0.999)	4	12.5 (>0.999)	1	0	0	mouthwash
0 (0.026)	0	25 (>0.999)	2	12/5 (>0.999)	1	62.5 (0.026)	5	injection

^*^P-value values were reported to compare the difference between the intervention groups and the negative control group

## Discussion

The use of drugs is still one of the most appropriate and commonly used methods in the treatment of different types of cancer. Different medical centers around the world are trying to find effective drugs with a selective effect on cancer cells along with a lesser effect on healthy cells. Among them, plant-derived agents like antioxidants are known as a great and promising source for the discovery of new drugs in this regard [17].

Arif et al. in their study in 2020 reported that the combination of ethanol obtained from two specific plants acts as ROS and it also has the potential of causing cancerous tissue in the studied rats. Accordingly, they attributed the main cause of cancer development to the accumulation of damage resulting from free radicals [18]. Similar to this study, in the current study, we used the theory stating that free radicals are the main cause of cancer, so we applied them to induce oral cancer in rats. Afterward, the significant results obtained in histopathological findings confirm that 4-Nitroquinoline-1-Oxide, as a free radical, can induce cancer.

In a meta-analysis published by Gloria et al. in 2020, the therapeutic and preventive effects of flavonoids (a type of antioxidant) on tongue cancer were discussed. In this regard, they reported that flavonoids could stop the cell cycle and prevent metastasis, as well as induce apoptosis in cancer cells. As well, since no side effects were seen, they introduced flavonoids as an attractive option for the treatment of tongue cancer [19]. Similarly, the results of our research show that the use of the antioxidant curcumin, as a preventive and therapeutic agent for oral cancer, can be very effective and efficient. Correspondingly, this substance inhibited the growth of KB cancer cells in the culture medium and caused no adverse effects on HUVEC cells in a concentration lower than 32 µg/ml. In this study, the KB cell line (human epithelial carcinoma cells) used as a model of cancer cells and HUVEC cells were used because the culture of oral epithelial cells is difficult, and in curcumin and SCC studies have been used previously [20]. According to Fig. 3, the toxicity of curcumin was time-dependently increased in 32 g/ml of curcumin and niosome-curcumin in normal cells. This is consistent with other evidence that turmeric caused a dose- and time-dependent induction of toxicity in several mammalian cell lines; and these alterations were observed at higher concentrations [21].

In the clinic, we also observed its preventive and inhibitory effects on the induced cancer in the rats that were similar to the results of the above-mentioned study.

In 2020, Sur et al. in their article mentioned Bitter Melon (a plant of the squash genus) as a very effective anti-cancer herb that can be used for the treatment of a variety of cancers, including the mouth, breast, prostate, skin, colon, and lung cancers. This has also been shown to strengthen the body’s immune system against diseases. They attributed all these properties to the antioxidant nature of this substance and its resistance to oxygen-free radicals [22]. In another study in 2019, Priyanka et al. showed that a substance called Maha Valley, used in traditional Indian medicine, as an antioxidant, significantly reduced 4-Nitroquinoline-1-oxide-induced oral cancer in rats [23]. In line with these two studies, the anti-cancer results obtained in our study, both at the microscopic and macroscopic levels, are regarding the antioxidant properties of curcumin. Curcumin counteracts the effects of 4-Nitroquinoline-1-oxide to cause cancer in the oral tissue, so it can be considered a successful anti-cancer. Also, previous studies indicate that curcumin has the possibility to inhibit tumor angiogenesis in SCC [20]. Another suggested mechanism for a prophylactic effect of curcumin is that it may inhibit the proliferation of OSCC cells via a specificity protein 1 and nuclear factor‑κB (NF‑κB) dependent mechanism. NF‑κB play important role in tumor cell survival, proliferation, and metastasis and exerts anti‑apoptotic properties on several types of cancer, including OSCC [24]. In the present study, we attempted to multiply this interaction using the niosome drug delivery system. Accordingly, a statistical analysis significantly indicated the success of this system, especially in the case of systemic use. Moreover, in the case of topical application, although the clinical difference among the groups was quite obvious, the result of the statistical analysis was not significant, which can be attributed to the small samples size. On the other hand, from the histopathological view, it was found that the rats that received injectable curcumin niosome had a better immune response compared to the other groups studied, and in general, the histopathological view of this group of the rats was very similar to that of the normal group and sometimes it was not possible to distinguish these two groups. These results confirm that systemic injections of curcumin may overcome poor oral bioavailability.

Mirzaei-Parsa et al. in a 2020 paper reported that the niosomal drug delivery system was significantly effective in the treatment of breast cancer [25]. Additionally, in a systematic review article published in 2020, Mehta described the niosomal drug delivery system as a new generation of drug carriers with a very wide domain. As well, he mentioned its use in the treatment of cancer as one of the important fields of this system and believed that this system has created an evolution in the drug delivery area, and this will subsequently result in the effective treatment of diseases with minimal side effects [26]. In addition, Akbarzadeh et al. in their study in 2020, concluded that the loading of letrozole, which is a drug used to treat breast cancer, into the niosomal system along with the functionalized folic acid and curcumin led to the drug stability for one month. On the other hand, it was shown that the drug inside the niosome can release the active substance in a wide pH range. Correspondingly, they considered this drug in combination can be effective on apoptosis and necrosis of breast cancer cells in two categories of MCF-7 and MDA-MB-231 [27]. Inconsistent with the above-mentioned three studies, in the present study, according to the initial results obtained from the culture medium of KB oral cancer cells, we found that the curcumin-loaded niosome is significantly effective in inhibiting the growth and necrosis of cancer cells compared with free curcumin. As well, based on the histopathological studies and statistical analyses, curcumin in the format of a niosome system was observed to be effective and efficient in inhibiting cancer induced in the rat’s mouth, and the use of a niosome system was one of the important reasons for these positive results.

It is recommended that further studies be performed in the following areas: evaluation of other sites such as the nasopharynx and esophagus, other types of nanoparticles such as nanoliposomes, and other antioxidants-loaded niosome.

## Conclusion

In the current study, at both cell culture and animal study stages, curcumin-loaded niosome was shown to be effective in preventing severe dysplasia. Curcumin-loaded niosome in the form of injection (as a systemic use) was shown to be more effective than the form of mouthwash (as a topical use). Additionally, this drug plays an effective role in improving and promoting the health of oral tissues.

## Data Availability

All data generated or analyzed during this study are included in this published article.

## Abbreviations

ACUC: Animal Care and Use Committee
ARRIVE: Animal Research: Reporting of In Vivo Experiments
DMEM: Dulbecco’s modified Eagle’s Medium
DMSO: Dimethyl sulfoxide
HUVEC: Human umbilical vein endothelial cells
NF‑κB: Nuclear factor‑κB
4NQO: 4-Nitroquinoline-1-Oxide
PAS: Periodic acid Schiff
RNS: Reactive nitrosative free radicals
ROS: Reactive oxygen species
SCC: Squamous cell carcinoma
